# *CBX7* gene expression plays a negative role in adipocyte cell growth and differentiation

**DOI:** 10.1242/bio.20147872

**Published:** 2014-09-04

**Authors:** Floriana Forzati, Antonella Federico, Pierlorenzo Pallante, Marianna Colamaio, Francesco Esposito, Romina Sepe, Sara Gargiulo, Antonio Luciano, Claudio Arra, Giuseppe Palma, Giulia Bon, Stefania Bucher, Rita Falcioni, Arturo Brunetti, Sabrina Battista, Monica Fedele, Alfredo Fusco

**Affiliations:** 1 Istituto di Endocrinologia ed Oncologia Sperimentale del CNR e/o Dipartimento di Medicina Molecolare e Biotecnologie Mediche, Università degli Studi di Napoli “Federico II”, 80131 Naples, Italy; 2 Dipartimento di Scienze Biomorfologiche e Funzionali, Universita' degli Studi di Napoli “Federico II”, 80131 Naples, Italy; 3 Istituto Nazionale dei Tumori, Fondazione Pascale, 80131 Naples, Italy; 4 Istituto Nazionale Tumori Regina Elena, Dipartimento di Oncologia Sperimentale, Laboratorio di Oncogenesi Molecolare, 00158 Rome, Italy; 5 Divisione di Chirurgia Plastica e Ricostruttiva, Istituto San Gallicano, 00144 Rome, Italy; 6 Istituto di Biostrutture e di Bioimmagini del CNR, 80145 Naples, Italy

**Keywords:** *Cbx7*, Adipose tissue, Mouse models

## Abstract

We have recently generated knockout mice for the *Cbx7* gene, coding for a polycomb group protein that is downregulated in human malignant neoplasias. These mice develop liver and lung adenomas and carcinomas, which confirms a tumour suppressor role for CBX7. The CBX7 ability to downregulate *CCNE1* expression likely accounts for the phenotype of the *Cbx7*-null mice.

Unexpectedly, *Cbx7-*knockout mice had a higher fat tissue mass than wild-type, suggesting a role of CBX7 in adipogenesis. Consistently, we demonstrate that *Cbx7*-null mouse embryonic fibroblasts go towards adipocyte differentiation more efficiently than their wild-type counterparts, and this effect is *Cbx7* dose-dependent. Similar results were obtained when *Cbx7*-null embryonic stem cells were induced to differentiate into adipocytes. Conversely, mouse embryonic fibroblasts and human adipose-derived stem cells overexpressing *CBX7* show an opposite behaviour.

These findings support a negative role of CBX7 in the control of adipocyte cell growth and differentiation.

## INTRODUCTION

CBX7 is a chromobox family protein component of a polycomb group (PcG) multiprotein PRC1-like complex, which is required to maintain the transcriptionally repressive state of many genes, including Hox genes, throughout development. PcG PRC1 complex acts *via* chromatin remodeling and modification of histones: it mediates monoubiquitination of histone H2A ‘Lys-119’, rendering chromatin heritably changed in its expressibility; promotes histone H3 trimethylation at ‘Lys-9’ (H3K9me3); binds to trimethylated lysine residues in histones, and possibly also other proteins (Schuettengruber et al., 2007; Wu et al., 2009; Scott et al., 2007). CBX7 has a critical role in the progression step of carcinogenesis. Indeed, a drastic downregulation of CBX7 expression has been reported by several groups in malignant neoplasias including thyroid (Pallante et al., 2008), pancreatic (Karamitopoulou et al., 2010), colon (Pallante et al., 2010), lung (Forzati et al., 2012), gastric (Jiang et al., 2010), bladder (Hinz et al., 2008), breast (Mansueto et al., 2010) carcinomas and a reduced growth rate is achieved by the restoration of CBX7 expression in carcinoma cells of different origin (Pallante et al., 2008; Pallante et al., 2010; Mansueto et al., 2010; Li et al., 2010). Moreover, a direct correlation between the loss of CBX7 expression and a more advanced stage of neoplastic disease and a poor survival has been frequently reported (Karamitopoulou et al., 2010; Pallante et al., 2010). The ability of CBX7 to positively or negatively regulate the expression of genes with a critical role in the epithelial–mesenchymal transition such as *CDH1* (E-cadherin) (Federico et al., 2009), *SPINK1*, *SPP1* (osteopontin, a gene involved in the metastatic process), *STEAP1* and others (Pallante et al., 2014) likely accounts for this correlation.

The tumor suppressor role of CBX7 has been very recently confirmed by the phenotype of *Cbx7*-null mice (Forzati et al., 2012). Indeed, these mice develop liver and lung adenomas and carcinomas, and accordingly, mouse embryonic fibroblasts (MEFs) derived from *Cbx7*-knockout (KO) embryos have a higher growth rate and a reduced susceptibility to senescence than their wild-type (WT) counterparts. Interestingly, we have also recently reported that CBX7 interacts with the High Mobility Group A1 (HMGA1) proteins, and that the two proteins have a competitory activity on the *CCNE1* promoter: HMGA1 activating and CBX7 inhibiting *CCNE1* promoter activity. We also suggest that overexpression of *CCNE1*, due to the lack of the negative control by CBX7, may be the main mechanism responsible for the neoplastic phenotype of the *Cbx7*-KO mice (Forzati et al., 2012).

The analysis of *Cbx7*-KO mice also showed a significant increase in fat tissue. This observation led us to investigate the role of CBX7 in the process of adipogenesis.

## RESULTS

### The *Cbx7*-null mice show an obese phenotype

**Fig. 1.**
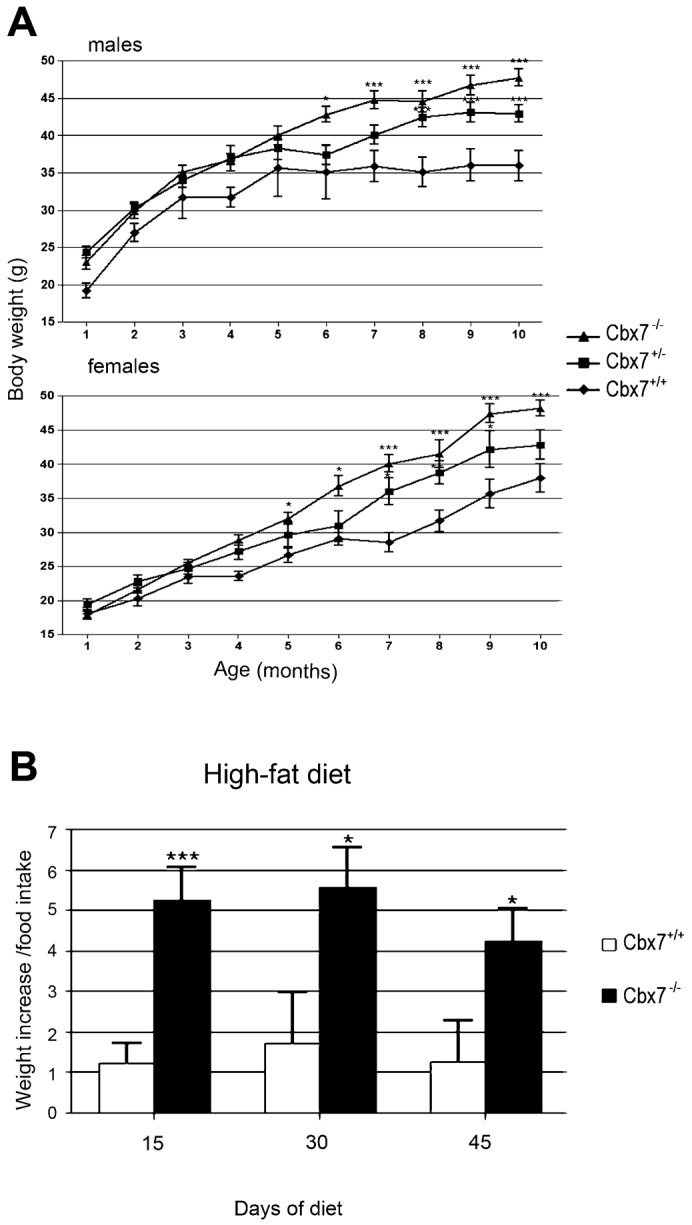
Body weight gain in Cbx7-KO mice. (A) Mean weights of cohorts of 10 *Cbx7^+/+^* (circle), 10 *Cbx7^+/−^* (square) and 10 *Cbx7^−/−^* (triangle) male and female mice as a function of age. *, *P*<0.05. **, *P*<0.01 ;***P<0.001 *vs Cbx7^+/+^* mice. (B) Weight gain/food intake ratio in cohorts of 8 *Cbx7^+/+^* and 8 *Cbx7^−/−^* male and female mice fed with a high-fat diet and analyzed at regular time intervals. *, *P*<0.05; **, *P*<0.01;***P<0.001.

The *Cbx7*-null mice were indistinguishable from their WT and heterozygous littermates until the fifth month of age, after which the body weight of both heterozygous and homozygous *Cbx7*-null mice (females and males) began to increase, raising up to 36% more than WT at 10 months (Fig. 1A).

**Fig. 2.**
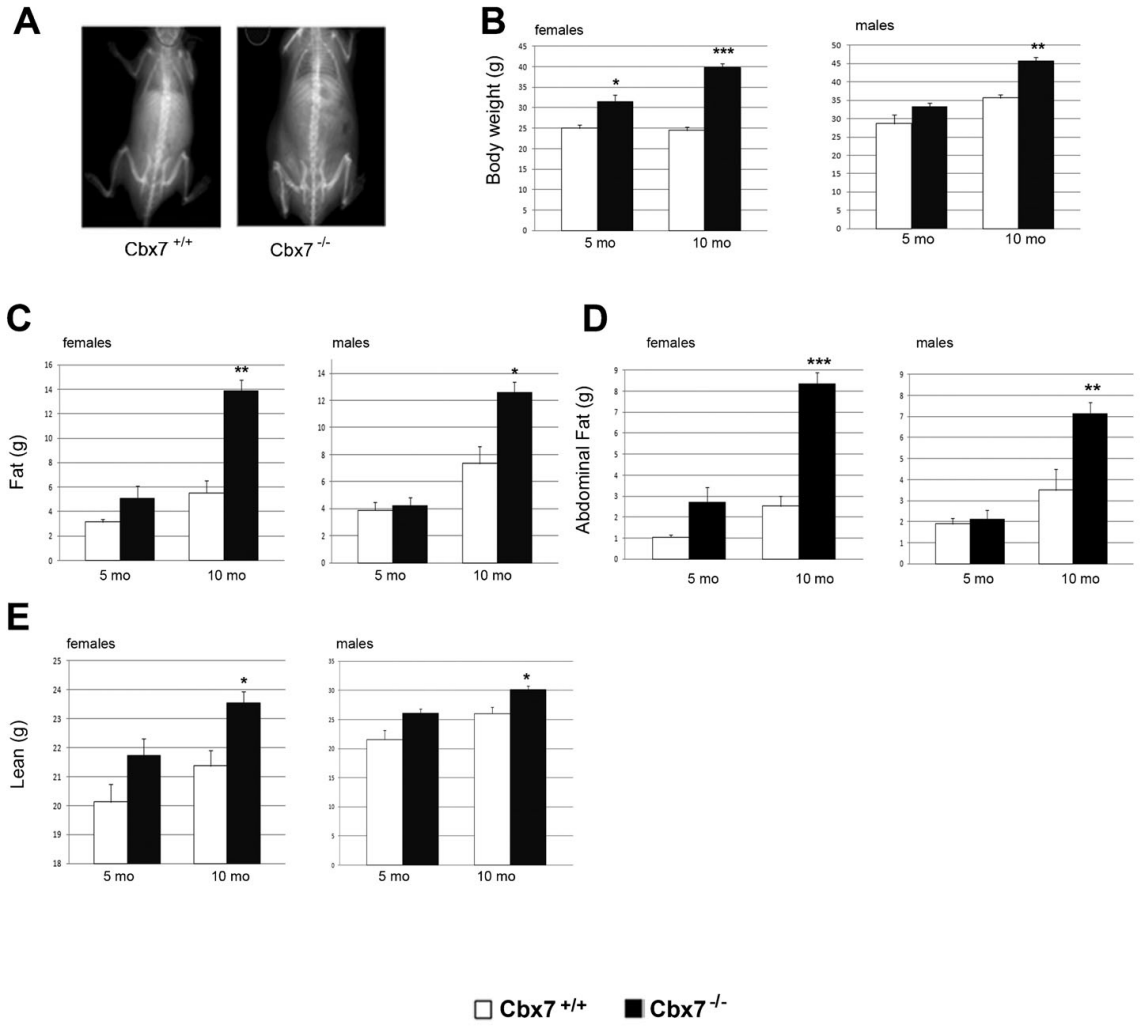
Increased body fat content and LBM in Cbx7-KO mice. (A) Representative DEXA image analyses in 10-month-old *Cbx7*^+/+^ and *Cbx7*^−/−^ mice. (B–E) Body weight, fat, abdominal fat and LBM content, as assessed by DEXA analyses, in 5-month-old and 10-month old *Cbx7*^+/+^ and *Cbx7*^−/−^ mice; *n* = 10. Values are expressed as means ± SEM. *P<0.05; **P<0.01; ***P<0.001.

Interestingly, *Cbx7*^−/−^ mutant mice challenged with a high-fat diet (HFD), shortly after weaning, gained weight more rapidly than WT control mice and earlier than standard diet (SD)-fed *Cbx7*^−/−^ mice (Fig. 1B). In fact, although the background strain is susceptible to weight gain starting from 4 weeks of HFD exposure, the body weight of all the HFD-fed *Cbx7*^−/−^ mice was already markedly increased even after only two weeks of diet with an average increase of 45%. Analysis of the food intake revealed that the *Cbx7*^−/−^ mice had a tendency to consume less food than their WT littermates (data not shown). Autoptic analysis of 10-month-old heterozygous and homozygous null mice, fed with a SD diet, revealed a significant increase in fat tissue, mainly at the abdominal region, which probably accounts for the increased weight gain. To better evaluate differences in body weight, fat and lean body mass (LBM), we examined cohorts of age- and sex-matched mice by dual-energy X-ray absorptiometry (DEXA). DEXA analysis showed significant differences (*P*<0.05) in the body weight of both female and male 10-month-old *Cbx7*^+/+^, *Cbx7*^+/−^ and *Cbx7*^−/−^ mice (Fig. 2A,B and data not shown). Similarly, the analysis of fat tissue confirmed an increased fat tissue in 10-month-old *Cbx7^−/−^* and *Cbx7^+/−^* mice than in their corresponding WT controls (Fig. 2C and data not shown). The overall difference in total fat mice between *Cbx7^+/+^*, *Cbx7*^+/−^ and *Cbx7*^−/−^ correlated with proportional differences in the amounts of intra abdominal fat (Fig. 2D and data not shown).

The LBM analysis was also significantly higher in *Cbx7*^−/−^ and *Cbx7*^+/−^ mice than in *Cbx7*^+/+^ mice at 10 months of age (Fig. 2E and data not shown).

### Downregulation of *Cbx7* expression plays a critical role in adipocyte differentiation *in vitro*

The results shown above suggested a critical role of *Cbx7* expression in adipocyte cell growth and/or differentiation. Therefore, we investigated whether the expression of *Cbx7* is regulated during adipocyte differentiation. As a model system we used the 3T3-L1 preadipocyte cells, which have been extensively characterized (Student et al., 1980). These cells undergo adipocyte conversion upon exposure to fetal bovine serum and the differentiating agents dexamethasone, methylisobutylxanthine and insulin (Student et al., 1980). Cells were harvested at various times during differentiation and both RNA and proteins were extracted.

**Fig. 3.**
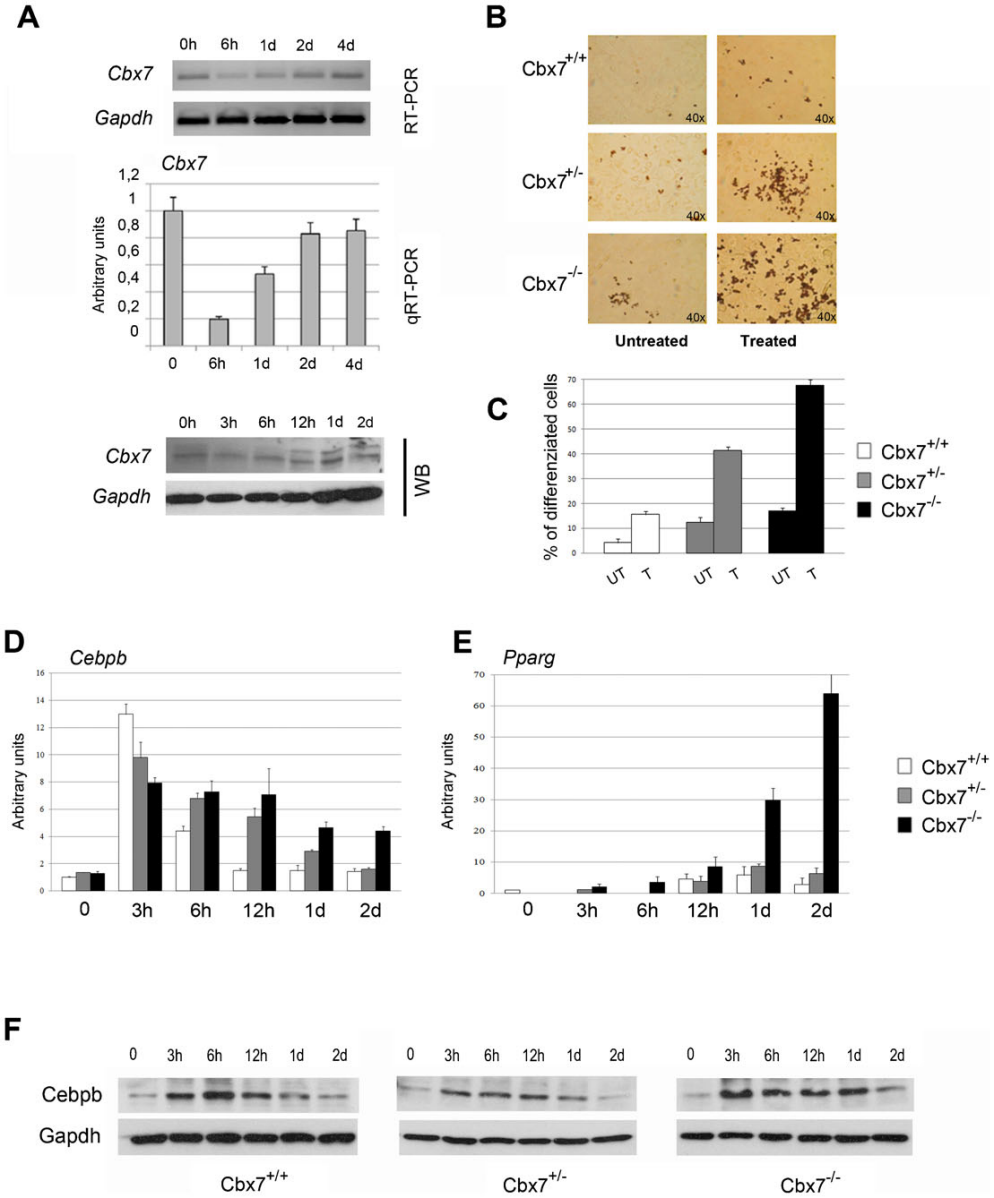
Cbx7 downregulation is required for adipocyte differentiation in 3T3-L1 and MEFs. (A) 3T3-L1 cells were treated with differentiating agents as described in Materials and Methods. Semi-quantitative (upper panel) and quantitative (middle panel) RT-PCR and Wester Blot analysis (lower panel) were performed at time 0 h, 6 h, 1 day (1 d), 2 days (2 d) and 4 days (4 d) from the beginning of hormone induction. (B) Lipid accumulation measured by Oil Red O staining in *Cbx7*^+/+^, *Cbx7*^+/−^ and *Cbx7*^−/−^ MEFs following adipocyte differentiation. Pictures were taken on a bright field microscope (Zeiss) at a magnification of 40×. (C) Quantification of the percent of cells stained in red (i.e., differentiated cells) in Cbx7^+/+^, Cbx7^+/−^ and Cbx7^−/−^ MEFs is expressed as mean ± SEM of three independent experiments. (D,E) Quantitative RT-PCR evaluating the expression of *Cebpb* and *Pparg* in *Cbx7*^+/+^, *Cbx7*^+/−^ and *Cbx7*^−/−^ MEFs during adipocyte differentiation at the indicated time points following hormone treatment. (F) Western blot analysis evaluating the protein level of Cebpb in *Cbx7*^+/+^, *Cbx7*^+/−^ and *Cbx7*^−/−^ MEFs during adipocyte differentiation.

Semiquantitative and quantitative RT-PCR analysis showed that endogenous *Cbx7* levels first decreased, between 6 h and 1 day after treatment with differentiating agents, and then returned to levels comparable to pretreatment (time zero) after 48 h (Fig. 3A, upper middle panel). Very similar results were obtained when Cbx7 protein expression was analyzed (Fig. 3A, lower panel).These findings indicate that the expression of *Cbx7* is regulated during adipocyte differentiation. Cell differentiation was verified by measuring the expression of various adipogenic markers, including CCAAT/enhancer binding protein beta (*Cebpb*), peroxisome proliferator-activated receptor gamma *(Pparg)* and adipocyte protein *(Ap2)* (data not shown).

To obtain additional evidence that *Cbx7* is involved in adipocyte differentiation, we isolated MEFs from *Cbx7*^+/+^, *Cbx7*^+/−^ and *Cbx7*^−/−^ embryos at 12.5 days post coitum (dpc), induced them to differentiate into adipocytes, as previously described (Hansen et al., 2002), and then stained them with Oil Red O, which is a marker of adipocyte differentiation. As shown in Fig. 3B,C, the number of cell clusters filled with lipid droplets (stained in red) was much higher in the *Cbx7*^−/−^ MEFs than in their WT counterparts, whereas *Cbx7*^+/−^ MEFs showed an intermediate behaviour. Accordingly, during the differentiating treatment, the expression level of *Cebpb* and *Pparg*, two adipocyte markers, differed in *Cbx7*^−/−^ and *Cbx7*^+/−^ MEFs *versus* WT controls (Fig. 3D–F). Indeed, the expression level of *Cebpb*, a transcription factor expressed during the early stages of adipogenesis (Farmer, 2006), increased after 3 h of differentiating treatment and, then, rapidly decreased after 6 h in WT MEFs. Conversely, *Cebpb* mRNA and proteins levels were slightly lower in *Cbx7*^−/−^ and *Cbx7*^+/−^ than in *Cbx7*^+/+^ MEFs, but remained stable even after 48 h (Fig. 3D,F).

Similarly, the expression of *Pparg*, a transcription factor involved in later stages of adipogenesis (Farmer, 2006), was much higher in *Cbx7*^−/−^ and *Cbx7*^+/−^ than in *Cbx7*^+/+^ MEFs (Fig. 3E).

### Cbx7-null embryonic stem cells undergo adipocyte differentiation with a higher efficiency compared to WT embryonic stem cells

**Fig. 4.**
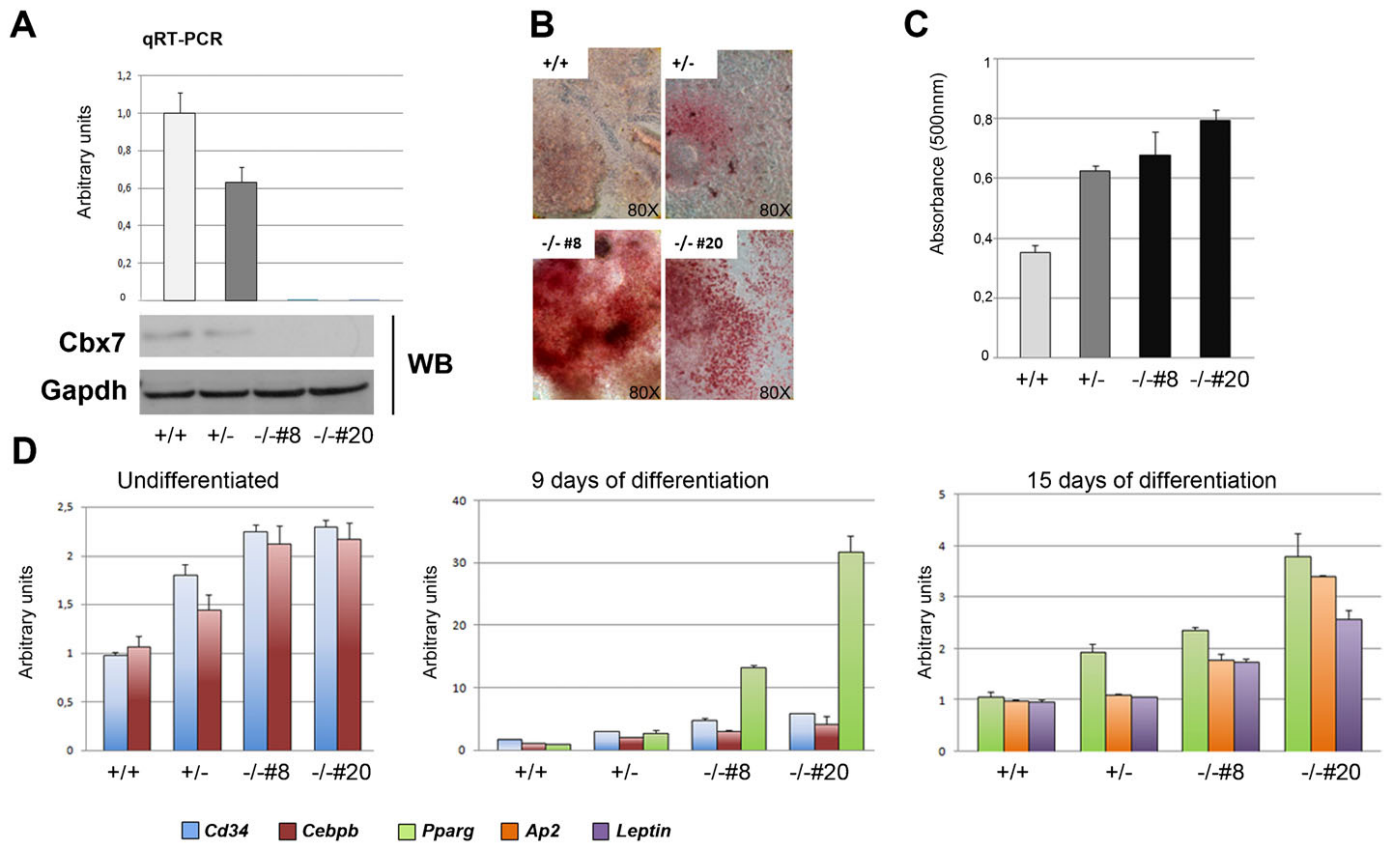
Increased adipocyte differentiation in Cbx7-KO ES cells. (A) Identification of *Cbx7^−/−^* clones by qRT-PCR (upper panel) and Western blot (lower panel) analyses. (B) Oil Red O staining of lipid droplets in *Cbx7*^+/+^, *Cbx7*^+/−^ and *Cbx7*^−/−^ ES-derived adipocytes. Pictures were taken on a bright field microscope (Zeiss) at a magnification of 80×. (C) Quantification of Oil Red O incorporation by measuring the absorbtion at 500 nm of Oil Red O extracted from cells shown in B. (D) qRT-PCR analyzing the expression of *Cd34*, *Cebpb*, *Pparg*, *Ap2* and *leptin* genes in ES cells before and after 9 and 15 days of adipocyte differentiation.

To better define the role of the Cbx7 protein in adipocyte differentiation, we generated *Cbx7*^−/−^ embryonic stem (ES) cells, starting from an already described (Forzati et al., 2012) *Cbx7*^+/−^ clone (Fig. 4A) and investigated their ability to undergo adipocyte differentiation (see Materials and Methods) compared to WT controls. After 20 days of treatment, embryoid bodies (EBs) were stained with Oil Red O to evaluate adipocyte differentiation. We observed a drastic increase in the ability to achieve adipogenesis in *Cbx7*^−/−^ compared to WT EBs, as determined by a drastic increase in the percentage of Oil Red O-positive *Cbx7*^−/−^ EBs and in the number of adipocytes *per* EB in *Cbx7*^−/−^ compared with WT EBs (Fig. 4B,C). Several *Cbx7*-null clones were analyzed for morphological differentiation and expression of adipocyte-specific markers by qRT-PCR assay. In particular, *Cd34*, an adipocyte precursor marker (Rodeheffer et al., 2008) and *Cebpb*, which is expressed early during adipocyte differentiation, were analyzed in undifferentiated ES cells and after 9 days of differentiation, whereas *Pparg*, which is expressed later, was analyzed after 9 and 15 days of differentiation. Finally, *Ap2* and *leptin*, which are the latest markers of the adipocyte differentiation, were analyzed only after 15 days of differentiation (Fig. 4D). The expression of all these genes is not detectable in WT undifferentiated ES cells, and induced after differentiation. Conversely, *Cd34* and *Cebpb* were already expressed in *Cbx7*^+/−^ and *Cbx7*^−/−^ untreated cells (Fig. 4D, left panel) and were significantly upregulated in differentiating EBs compared with WT controls (Fig. 4D, middle panel). Similarly, *Pparg* was strongly upregulated in *Cbx7*^−/−^ (and to a lesser extent also in *Cbx7*^+/−^) EBs at 9 days of differentiation compared to WT controls, and similar expression levels were observed in both *Cbx7*^+/−^ and *Cbx7*^−/−^ EBs also at 15 days of differentiation. Finally, the increase of *Ap2* and *leptin* expression at 15 days of differentiation was significantly higher in *Cbx7*^−/−^ EBs compared to controls (Fig. 4D, right panel).

Therefore, these results confirm that the loss of *Cbx7* gene expression enhances adipocyte differentiation.

### Impairment of adipocyte differentiation in Cbx7-overexpressing MEFs

**Fig. 5.**
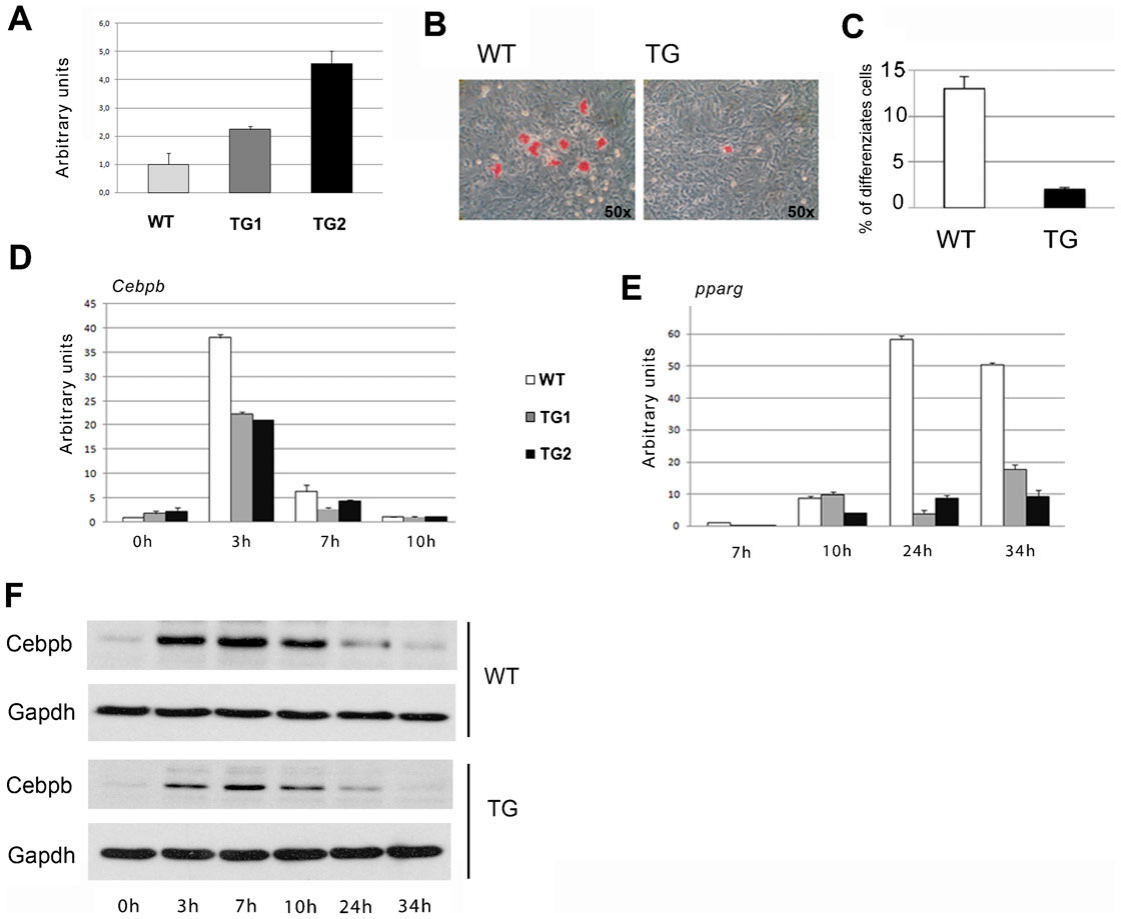
Overexpression of Cbx7 inhibits MEF adipocyte differentiation. (A) qRT-PCR showing the expression levels of *Cbx7* gene in MEFs derived from two Cbx7 transgenic lines (TG1 and TG2) and control WT mice. (B) Lipid accumulation measured by Oil Red O staining in MEFs as in A, following adipocyte differentiation. Pictures were taken on a bright field microscope (Zeiss) at a magnification of 50×. (C) Quantification of the percent of cells stained in red (i.e., differentiated cells) in WT, TG1 and TG2 MEFs is expressed as mean ± SEM of two independent experiments. (D,E) Quantitative RT-PCR evaluating the expression of *Cebpb* and *Pparg* gene in WT, TG1 and TG2 MEFs during adipocyte differentiation at the indicated time points following hormone treatment. (F) Western blot analysis evaluating the protein level of Cebpb in WT and TG1 MEFs during adipocyte differentiation.

As a further control of the negative role of CBX7 in adipocyte differentiation, we isolated MEFs from two mouse transgenic lines (TG-Cbx7-1 and TG-Cbx7-2), overexpressing the *Cbx7* gene under the transcriptional control of a cytomegalovirus promoter (Fig. 5A) (Forzati et al., 2012), and evaluated their ability to differentiate into adipocytes. Oil Red O staining showed that the TG-Cbx7 MEFs have a lower rate of adipocyte differentiation with respect to WT MEFs (Fig. 5B,C). Consistently, significant reduction in *Cebpb* mRNA and protein levels was observed in MEFs overexpressing *Cbx7* after 7 h of the differentiating treatment (Fig. 5D,F). Also *Pparg* expression, evaluated by qRT-PCR, was much lower in the TG-Cbx7 MEFs at 24 and 34 hours in comparison with the WT controls (Fig. 5E).

### CBX7 expression has inhibitory activity on adipocyte differentiation of human adipose-derived stem cells

**Fig. 6.**
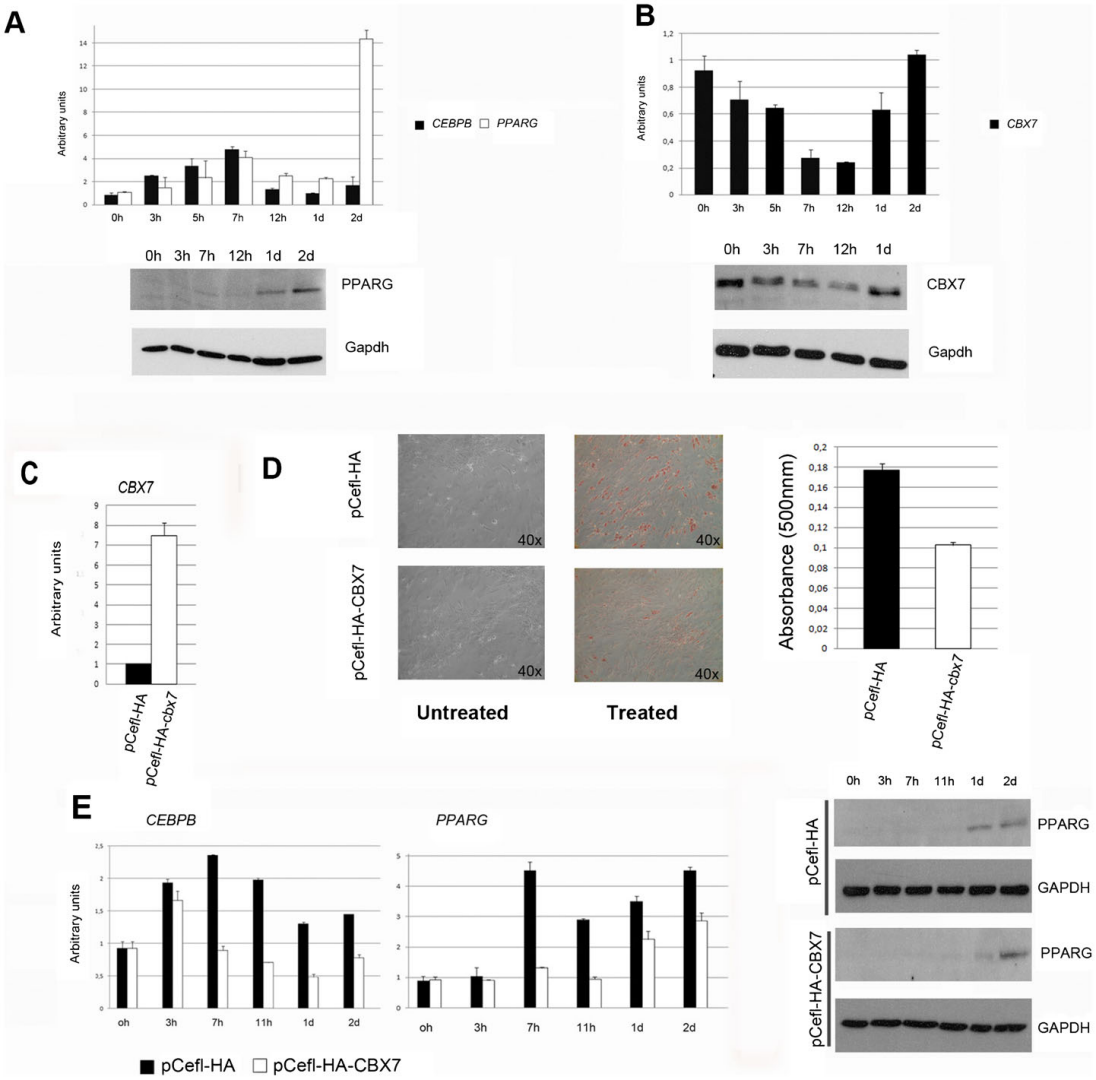
CBX7 expression in adipocyte differentiation of adipose-derived stem cells. (A) The adipose-derived stem cells, ADS1, were analyzed for the capability to differentiate towards the adipocytic lineage. After treatment with differentiating agents, as described in Materials and Methods, cells were harvested at time 0 h, 3 h, 5 h, 7 h, 12 h, 1 day (1 d), and 2 days (2 d) from the beginning of hormone induction and RNAs were analyzed for the expression of two different marker of differentiation as *CEBPB* and *PPARG*, by qRT-PCR and Western blot. (B) mRNA and protein level of *CBX7* was analyzed by qRT-PCR and Western blot, in ADS1 differentiated cells, at the indicated time points following hormone treatment. (C) qRT-PCR analysis of CBX7 expression in human adipose-derived stem cells, ADS1, transfected with the empty vector (pCEFL-HA) or a vector expressing CBX7 (pCEFL-HA CBX7). The value of the control is assumed equal to 1. (D) Oil Red O staining of lipid droplets in empty vector or Cbx7 transfected ADS cells and Quantification of Oil Red O incorporation by measuring the absorption at 500 nm. Pictures were taken on a bright field microscope (Zeiss) at a magnification of 40×. (E) Western blot and Quantitative RT-PCR evaluating the expression of *CEBPB* and *PPARG*, in CBX7- and empty vector- ADS1 transfected cells, during adipocyte differentiation at time 0 h, 3 h, 7 h, 12 h, 1 day (1 d), and 2 days (2 d) following hormone treatment. A representative experiment is reported.

To verify whether CBX7 has a role in adipocyte differentiation also in humans, we focused our studies on two human adipose-derived stem (ADS) cells, named ADS1 and ADS3 cells. ADS cells have been demonstrated to be able to differentiate in adipocyte (Folgiero et al., 2010) (Fig. 6A; supplementary material Fig. S1A). We first evaluated the expression of CBX7 during the adipocyte differentiation of these cells. As shown in Fig. 6B (and supplementary material Fig. S1B), CBX7 downregulation is observed in both ADS1 and ADS3 cells after few hours of the differentiating treatment, as it occurs for the murine 3T3L1 cells (Fig. 3A). Then, the *CBX7* mRNA and protein levels return to those comparable to pretreatment levels in terminally differentiated adipocytes (Fig. 6B; supplementary material Fig. S1B).

Subsequently, to verify that CBX7 is a negative regulator of differentiation we transfected the adipose-derived stem cells with CBX7 (Fig. 6C; supplementary material Fig. S1C). The overexpression of CBX7 resulted in altered adipocyte differentiation. In fact, the Oil Red O staining showed that the cells overexpressing CBX7 have a lower rate of adipocyte differentiation with respect to control cells (Fig. 6D). Consistenly, after the differentiating treatment the CEBPB and PPARG levels are lower in the cells overexpressing CBX7 compared to the control cells at the same time points (Fig. 6E,F; supplementary material Fig. S1D).

## DISCUSSION

The polycomb group (PcG) proteins form chromatin-modifying complexes that are essential for embryonic development and stem cell renewal and are commonly deregulated in cancer (Bracken et al., 2006). CBX7 is a chromobox family protein and a member of the polycomb repressive complex 1 (PRC1). We have recently generated *Cbx7*-KO mice validating a tumor suppressor role of the *Cbx7* gene since these mice developed liver and lung adenomas and carcinomas that were associated with an increased expression of the *cyclin E* gene. However, *Cbx7*-KO mice also showed a significantly higher amount of fat tissue with respect to WT. These findings suggested a negative role of the *Cbx7* gene in the control of adipocyte cell proliferation and differentiation prompting us to investigate the role of *Cbx7* in adipogenesis. Several experiments reported here seem to confirm this suggestion. Indeed, MEFs and ES cells null for *Cbx7* go towards adipocyte differentiation much more efficiently than their WT counterparts, and this effect is *Cbx7* dose-dependent since MEFs and ES cells carrying only one impaired *Cbx7* allele show an intermediate behaviour. Moreover, MEFs overexpressing *Cbx7* showed a lower ability to differentiate to adipocytes with respect to the WT MEFs. Interestingly, we have been able to confirm the negative role of CBX7 in adipogenic differentiation also in human adipose-derived stem cells. Indeed, we show that the differentiation of these cells results in an immediate downregulation of CBX7 expression, whereas CBX7 overexpression has an inhibitory effect on their adipogenic differentiation.

Since we have previously shown that CBX7 interacts with HMGA1 (Forzati et al., 2012) we hypothesize that the negative role of CBX7 in adipocyte differentiation occurs *via* its antagonistic effect on HMGA1. Therefore, the mechanism would be very similar to that already described for the regulation of the *CCNE1* gene, where the HMGA1 positive regulation is antagonized by the opposite effect exerted by CBX7 (Forzati et al., 2012). Consistently, our preliminary results demonstrate that CBX7 inhibits the HMGA1-induced positive regulation of the *OB* gene, and chromatin immunoprecipitation (ChIP) assays and functional analyses demonstrate that both proteins are able to bind the *OB* gene promoter with opposite competitive effects on its activity. Interestingly, HMGA1 and CBX7 act in an opposite manner in human malignancies, *HMGA1* overexpression and *CBX7* downregulation correlating with a poor prognosis (Pallante et al., 2008, Pallante et al., 2010; Karamitopoulou et al., 2010; Fusco and Fedele, 2007; Fedele and Fusco, 2010). Since we have previously shown that HMGA1 is able to negatively regulate CBX7 expression (Mansueto et al., 2010) and that HMGA1 positively regulates miR-181 that has CBX7 as target, we can envisage a HMGA1-CBX7 network that operates in the regulation of tumour progression and adipocyte cell growth and differentiation. Interestingly, a drastic increase in miR-181b expression has been observed in 3T3-L1 cells after induction of adipocyte differentiation (R.F., personal communication).

A role of a member of the polycomb gene protein family in adipogenesis is not surprising since previous studies have shown that Ezh2 and its H3K27 methyltransferase activity are required for adipogenesis. Ezh2 directly represses *Wnt1*, -*6*, -*10a*, and -*10b* genes in preadipocytes and during adipogenesis. Deletion of Ezh2 eliminates H3K27me3 on *Wnt* promoters and derepresses *Wnt* expression, which leads to activation of Wnt/β-catenin signaling and inhibition of adipogenesis. Ectopic expression of the WT Ezh2, but not the enzymatically inactive F667I mutant, prevents the loss of H3K27me3 and the defective adipogenesis in *Ezh2*^−/−^ preadipocytes (Wang et al., 2010). Moreover, the involvement of the polycomb family protein in adipogenic differentiation seems also supported by data reporting histone H3 modifications associated with differentiation and long-term culture of mesenchymal adipose stem cells. Indeed, it has been shown that adipogenic differentiation at early passage results in H3K27 demethylation and H3K9 acetylation specifically on adipogenic promoters, whereas at late passages transcriptional upregulation is impaired, H3K27 trimethylation is maintained and H3K9 acetylation is inhibited on the adipogenic promoters implicating a polycomb-mediated epigenetic program regulating adipogenesis (Noer et al., 2009).

In this context, the results reported here would suggest the participation of also the polycomb family member CBX7, in the control of adipocyte cell growth and differentiation likely acting with an opposite role with respect to Ezh2 (Noer et al., 2009).

## MATERIALS AND METHODS

### Generation and genotyping of mutant mice

The generation and genotyping of the *Cbx7*^+/−^, *Cbx7^−/−^* and transgenic (TG-*Cbx7*) mice has been already described (Forzati et al., 2012). All mice were maintained under standardized nonbarrier conditions in the Laboratory Animal Facility of Istituto dei Tumori di Napoli (Naples, Italy) and all studies were conducted in accordance with Italian regulations for experimentations on animals.

### Cell cultures

Primary MEFs from *Cbx7*^+/+^, *Cbx7*^+/−^, *Cbx7^−/−^* and TG-*Cbx7* mice, were obtained from 12.5 dpc embryos following standard procedures, as previously described (Forzati et al., 2012). They were grown in Dulbecco's modified Eagle's medium (DMEM) (Life Technologies, Grand Island, NY) containing 10% fetal bovine serum (FBS) (Hyclone, South Logan, Utah), 1% glutamine (Life Technologies), 1% penicillin/streptomycin and 1% gentamicin (Life Technologies) (Forzati et al., 2012).

The mouse NIH 3T3-L1 cells used in this study were generously donated by Dr. E. Santos (National Cancer Institute, NIH, Bethesda MD). Cells were grown in DMEM supplemented with 10% calf serum (CS) (Life Technologies).

ES cells were grown on a layer of mitomycin C-inactivated primary fibroblasts in DMEM, 15% FBS (Euroclone, Pero (MI) Italy), 2 mM glutamine (Life Technologies), 1 mM sodium pyruvate (Life Technologies), 1× non-essential amino acids (Life Technologies), 50 ug/ml penicillin/streptomycin (Life Technologies), 1000 units/ml leukemia inhibitory factor (LIF, Chemicon), and 0.1 mM β-mercaptoethanol. Medium was changed daily, and ES cells were split every 2–3 days (Dani et al., 1997). ES cells homozygous for the *Cbx7*-null allele (*Cbx7* double-KO) were obtained by selecting *Cbx7^+/−^* ES cells, previously described (Forzati et al., 2012) with high geneticin concentration (Life Technologies) (12 mg/ml). Among 20 clones obtained and screened by Southern blot as previously described (Forzati et al., 2012), two resulted *Cbx7* double-KO.

Human embryonic kidney HEK 293 cells were grown in DMEM containing 10% FBS, 1% glutamine and 1% penicillin/streptomycin.

The ADS cells were grown in DMEM/F12 supplemented with 20% FBS (Hyclone), 1% antibiotics and 1% fungizone (Life Technologies) (Folgiero e al., 2010).

### Adipocyte differentiation and Oil Red O staining

Adipocyte differentiation was induced in *Cbx7^+/+^*, *Cbx7*^+/−^, *Cbx7^−/−^* and TG-*Cbx7* MEFs, as well as in 3T3-L1 pre-adypocytic cells. Induction of adipocyte differentiation was performed essentially as described elsewhere (Student et al., 1980). Briefly, cells were grown in their medium until confluence. Two days later, the medium was supplemented with 0.5 mM 1-methyl-3-isobutylxanthine, 10^−6^ M dexamethasone and 10 mg/ml insulin for 48 h. Cells were cultured for a further 6 days more in the same medium but without dexamethasone and methylisobutylxanthine. After 20 days of differentiation treatment, the culture medium was removed, and cells were washed twice with phosphate buffer saline (PBS) and fixed with 4% formalin/PBS. Cells were then washed three times with PBS, incubated in 60% isopropyl alcohol for 10 min and stained with 1.8% Oil Red O (Sigma St Louis, MO) in 60% isopropyl alcohol for 10 min. After three more washings with deionized water, cells were scored for adipocyte differentiation and photographed.

ES cells were induced to differentiate as described (Dani et al., 1997). Briefly, hanging drops containing 10^3^ cells in 20 µl culture medium were maintained for 2 days under the lid of bacteriological dishes filled with PBS. The EBs formed were then transferred into bacteriological plates and maintained for 3 days in suspension in cultivation medium supplemented either with 0.1% DMSO or with all-*trans*-retinoic acid (ATRA) (10^−8^ µM). Medium was changed every day. EBs were maintained 2 days more in suspension in cultivation medium and then were allowed to settle onto gelatin-coated plates in the presence of differentiation medium. This medium consists of cultivation medium supplemented with 85 nM insulin, 2 nM triiodothyronine, and 10% selected fetal calf serum (Life Technologies). Medium was changed every 2 days (Dani et al., 1997). EBs were harvested for RNA extraction after 0, 9, and 15 days of differentiation. Alternatively, after 16 days, EBs were stained with Oil Red O to assess adipocyte differentiation. In order to exactly quantify the Oil Red O staining, samples were de-stained with 100% isopropanol for 10 minutes. The absorbance of the eluates was measured at 500 nm in microplate reader (LX 800, Universal Microplate Reader, BioTek, Winooski, VT). 100% isopropanol was used as blanc.

Human ADS cells were induced to differentiate as described (Folgiero et al., 2010). Briefly, cells were grown in their medium until confluence. Then, 0.5 µM 1-methyl-3-isobutylxanthine, 1 µM dexamethasone, 10 µg/ml insulin, and 100 µM indomethacin were added to the culture medium. The Oil Red O staining was performed as described above.

### Isolation of mRNA and RT-PCR assays

Total RNA was extracted using TRI-reagent solution (Invitrogen, Carlsbad, CA,) according to the manufacturer's protocol. cDNA was synthesized from total RNA using random hexamers (100 mM) and MuLV reverse transcriptase (Perkin Elmer, Santa Clara, CA). Semi-quantitative PCRs were run with a 25 µl volume for 25 cycles (30 sec at 94°C, 30 sec at 55°C, and 30 sec at 72°C) using the Gene Amp PCR System 9700 (Applied Biosystems, Foster City, CA). RNA samples that had not been reverse transcribed before PCR served as negative control. Quantitative RT-PCR was performed with the SYBR Green PCR Master Mix (Applied Biosystems) under the following conditions: 10 min at 95°C followed by 40 cycles (15 sec at 95°C and 1 min at 60°C). Subsequently, a dissociation curve was run to verify amplification specificity. Each reaction was performed in triplicate. We used the 2^−ΔCT^ method to calculate the relative expression levels (Livak and Schmittgen, 2001). The following primer sequences were used to amplify the indicated mouse genes: *Cbx7*: 5′-ttgtcatggcctacgagga-3′ (forward) and 5′-tgggtttcggacctctctt-3′ (reverse); *Pparg:* 5′-tgctgttatgggtgaaactctg-3′ (forward) and 5′-ctgtgtcaaccatggtaatttctt-3′ (reverse); *Cebpb*: 5′-tctactacgagcccgactgc-3′ (forward) and 5′-ggtaggggctgaagtcgat-3′ (reverse); *G6pd*: 5′-gaaagcagagtgagcccttc-3′ (forward) and 5′-cataggaattacgggcaaaga-3′ (reverse) and the human ones *CBX7*: 5′-cgagtatctggtgaagtggaaa-3′ (forward) and 5′-gggggtccaagatgtgct-3′ (reverse); *PPARG:* 5′-aaggccattttctcaaacga-3′(forward) and 5′-tcaaaggagtgggagtggtc3′ (reverse); *CEBPB:* 5′-gcaagagccgcgacaag-3′ (forward) and 5′-ggctcgggcagctgctt-3′ (reverse); *G6PD*: 5′-gatctaccgcatcgaccact-3′ (forward) and 5′-agatcctgttggcaaatctca-3′ (reverse).

### Body weight growth curves, length and DEXA analysis

The mice were generally kept and bred with *ad libitum* access to water and pelleted standard food (Mucedola, Milano, Italy). The body weight was measured monthly, beginning at 4 weeks of age, for ten months. Body length was measured by manual immobilization, extension and measurement of the nasal–anal length. Analysis of body fat and LBM in *Cbx7*^−/−^, *Cbx7*^+/−^, and WT controls was performed by DEXA using the Lunar PIXImus Mouse Densitometer (Wipro GE Healthcare, Madison, WI) (Nagy and Clair, 2000). Before analysis, mice were anesthesized with isoflurane (isoflurane 1.5% plus 2 L/min oxygen).

### High- and low-fat diet studies

Soon after weaning, cohorts of 8 *Cbx7^+/+^* and *Cbx7^−/−^* mice were provided with either a low-fat diet (LFD) containing 10% fat or a high-fat diet (HFD) containing 45% fat (Mucedola) for up to 45 days. Body weight and food intake were monitored every 15 days.

### Plasmids, transfections

Expression vectors coding for CBX7 (Federico et al., 2009), or the empty vectors were transfected into human ADS cells using Neon Electroporation System (Invitrogen) according to manufacturer's instructions, and neomycin resistant mass cell populations were obtained.

### Protein extraction and Western blotting

Protein extraction and Western blotting were carried out as reported elsewhere (Forzati et al., 2012). The antibodies used were: anti-GAPDH (sc-32233, Santa Cruz, Biotechnology, Inc., Santa Cruz, CA), anti-CEBPB (sc-150, Santa Cruz), anti-PPARG (2443-Cell Signaling Technology, Inc., Danvers, MA)), anti-CBX7 (sc-481, Santa Cruz).

### Statistical analyses

We used the Student's *t*-test for intergroup comparisons. The Kruskall–Wallis test, and Dunn and Bonferroni post hoc analyses were used to compare DEXA results. The statistical analyses were performed using GraphPad Prism v.6.0 (La Jolla, CA, USA). A *P* value<0.05 was considered statistically significant.

## Supplementary Material

Supplementary Material
