# Supplementary material for: CBX7 gene expression plays a negative role in adipocyte cell growth and differentiation
Source: Biol Open. 2014 Sep 4;3(9):871–9. doi: 10.1242/bio.20147872 (PMC4163664; doi:10.1242/bio.20147872)
Supplement: Supplementary Material [file supp_3_9_871__index.html]

CBX7 gene expression plays a negative role in adipocyte cell growth and differentiation — CBX7 gene expression plays a negative role in adipocyte cell growth and differentiation — Supplementary Material 

# *CBX7* gene expression plays a negative role in adipocyte cell growth and differentiation

## bio.20147872 Supplementary Material

**Files in this Data Supplement:**

- Supplementary Material - Floriana Forzati et al. doi: 10.1242/bio.20147872
