# Supplementary material for: CBX7 gene expression plays a negative role in adipocyte cell growth and differentiation
Source: Biol Open. 2014 Sep 4;3(9):871–9. doi: 10.1242/bio.20147872 (PMC4163664; doi:10.1242/bio.20147872)
Supplement: Supplementary Material [file supp_bio.20147872_bio.20147872-s1.pdf]

Biology Open (2014) 000, 1–9 doi:10.1242/bio.20147872

Supplementary Material
Floriana Forzati et al. **doi: 10.1242/bio.20147872**

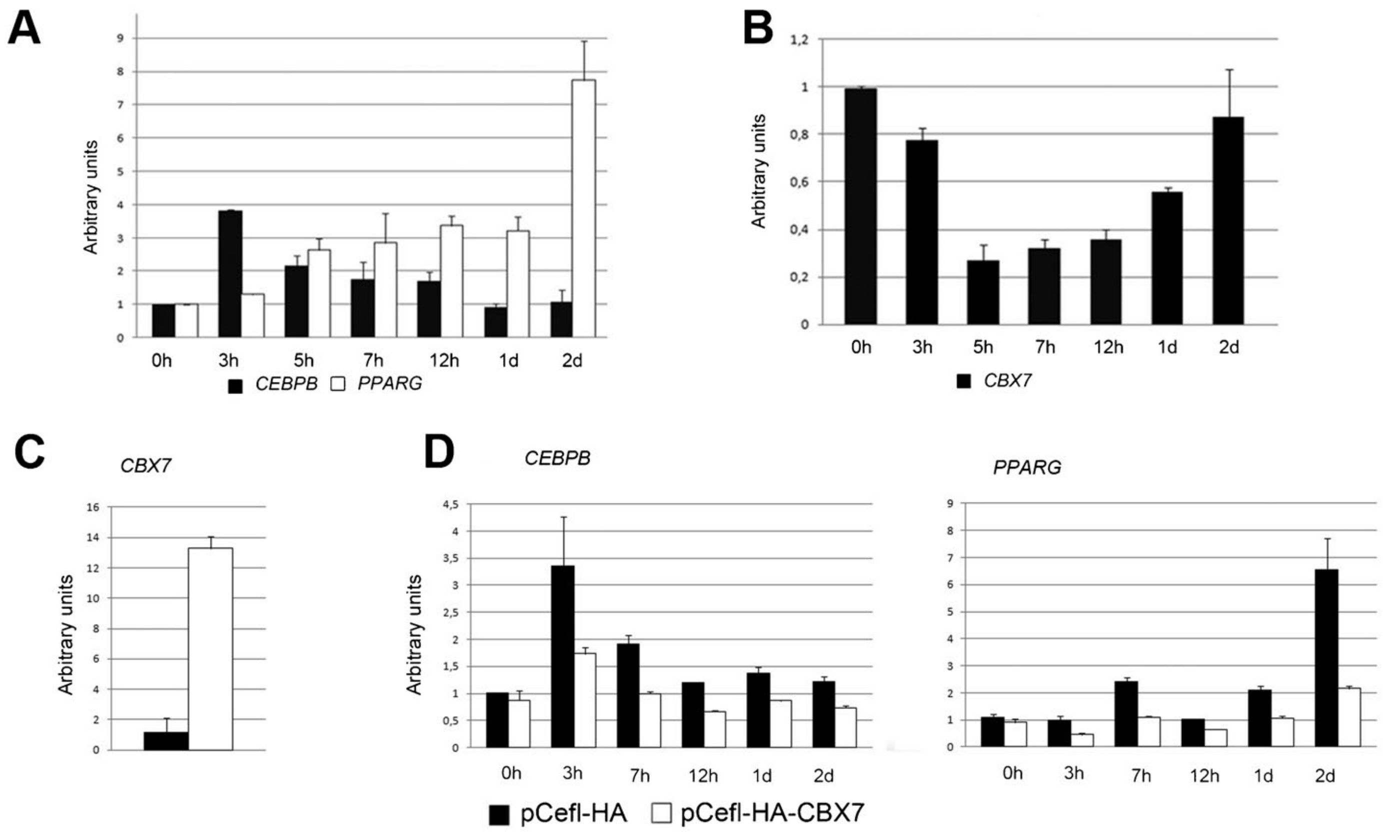

Fig. S1. CBX7 expression in adipocyte differentiation of adipose-derived stem cells. (A) The adipose-derived stem cells, ADS3, were analyzed for the capability to differentiate towards the adipocytic lineage. After treatment with differentiating agents, as described in Materials and Methods, cells were harvested at time 0 h, 3 h, 5 h, 7 h, 12 h, 1 day (1 d), and 2 days (2 d) from the beginning of hormone induction and RNAs were analyzed for the expression of two different marker of differentiation as *CEBPB* and *PPARG*, by qRT-PCR. (B) Expression level of *CBX7* was analyzed by qRT-PCR, in ADS3 differentiated cells, at the indicated time points following hormone treatment. (C) qRT-PCR analysis of CBX7 expression in human adipose-derived stem cells, ADS3, transfected with the empty vector (pCEFL-HA) or a vector expressing CBX7 (pCEFL-HA CBX7). The value of the control is assumed equal to 1. (D) Quantitative RT-PCR evaluating the expression of *CEBPB* and *PPARG*, in CBX7- and empty vector- ADS3 transfected cells, during adipocyte differentiation at time 0 h, 3 h, 7 h, 12 h, 1 day (1 d), and 2 days (2 d) following hormone treatment. A representative experiment is reported.
